# Association of Interleukin Genes *IL10* and *IL10RB* with Parameters of Overweight in Military Students

**DOI:** 10.3390/genes13020291

**Published:** 2022-02-01

**Authors:** Ewelina Maculewicz, Magdalena Dzitkowska-Zabielska, Bożena Antkowiak, Oktawiusz Antkowiak, Andrzej Mastalerz, Aleksandra Garbacz, Myosotis Massidda, Aleksandra Bojarczuk, Łukasz Dziuda, Paweł Cięszczyk

**Affiliations:** 1 Faculty of Physical Education, Jozef Pilsudski University of Physical Education in Warsaw, 00-809 Warsaw, Poland; andrzej.mastalerz@awf.edu.pl; 2 Military Institute of Hygiene and Epidemiology, 01-163 Warsaw, Poland; bozena.antkowiak@wihe.pl (B.A.); oktawiusz.antkowiak@wihe.pl (O.A.); 3 Faculty of Physical Education, Gdansk University of Physical Education and Sport, 80-336 Gdansk, Poland; magdalena.dzitkowska@gumed.edu.pl (M.D.-Z.); aleksbojar@gmail.com (A.B.); pawel.cieszczyk@awf.gda.pl (P.C.); 4 Warsaw University of Life Sciences—SGGW, 02-787 Warsaw, Poland; aleksandra_garbacz@sggw.edu.pl; 5 Sport and Exercise Sciences Degree Courses, Faculty of Medicine and Surgery, University of Cagliari, 09100 Cagliari, Italy; myosotis.massidda@unica.it; 6 Military Institute of Aviation Medicine, 01-755 Warszawa, Poland; ldziuda@wiml.pl

**Keywords:** Interleukin, *IL10*, *IL10RB*, genetic polymorphisms, haplotype, BMI, fat percentage, FMI

## Abstract

Background: To date, nearly 300 single-nucleotide polymorphisms (SNPs) associated with BMI, waist-to-hip ratio, and other adiposity traits have been identified by GWAS. With regards to *IL10*, at least 49 *IL10*-associated polymorphisms have been reported. However, little is known regarding the relationship between SNPs of the *IL10* gene and the risk of obesity in young men. The aim of the present study was to investigate the relationship between SNPs of the *IL10* and *IL10RB* genes and the risk of obesity in young men. Methods: A cohort of 139 male students were enrolled and the following *IL10* and *IL10RB* SNPs were analyzed: *IL10* (rs1518110), *IL10* (rs3024491), *IL10RB* (rs2834167). The subjects were divided into groups depending on obesity parameters: body mass index (BMI), fat mass index (FMI) and fat percentage (Fat%). Statistical analysis was conducted for a single locus and haplotypes, an association between SNPs and body composition parameters was tested with four genetic models: dominant, recessive, codominant and overdominant mode of inheritance (MOI). Results: Significant association was found for interaction *IL10* (rs1518110) × *IL10RB* (rs2834167) with Fat% value exceeding 20 in codominant (*p*-value = 0.03, OR = 0.34, 95% CI 0.08 1.44) and dominant model (*p*-value = 0.03, OR = 0.34, 95% CI 0.08 1.44) Conclusion: Our study shows for the first time that there is a correlation between the occurrence of specific polymorphisms of *IL10* gene (rs1518110, rs3024491 and rs2834167) and the possibility of obesity.

## 1. Introduction

In recent years, in the majority of fully developed countries of the world, a constant and noticeable increase in the frequency of the occurrence of overweight and obesity has been observed. According to the American Heart Association, obesity is the cause of many adverse changes in the body that are directly proportional to health disorder development [1,2].

Obesity is often associated with chronic low-grade inflammation. Inflammation is a physiological response necessary to restore homeostasis altered by diverse stimuli. However, excessive inflammatory signals might have deleterious effects. Such triggers also occur in obesity. Faloia et al. particularly emphasized that inflammation-associated intracellular signalling pathways linking inflammation and obesity exist. Indeed, studies in mice and humans have shown that nutrients consumption might acutely evoke inflammatory responses. It is believed that the inciting signal of inflammation is overfeeding. This initiates a cascade of signalling pathways [3].

Obesity is a pro-inflammatory condition in which hypertrophied adipocytes and adipose tissue-resident immune cells (primarily lymphocytes and macrophages) both contribute to increased circulating levels of pro-inflammatory cytokines [4].

Adipose tissue in lean conditions releases anti-inflammatory adipokines such as adiponectin, also transforming growth factor β (TGFβ), interleukin (IL) 10, IL4, IL13, IL1 receptor antagonist (IL1RA), and apelin. Compared with lean individuals, obese adipose tissue mainly secretes pro-inflammatory cytokines, i.e., TNFα, IL6, leptin, visfatin, resistin, angiotensin II, and plasminogen activator inhibitor 1 [5]. It has been previously demonstrated that circulating levels of anti-inflammatory cytokine, *IL10* are elevated in obese patients [6,7,8] while low *IL10* levels are associated with the metabolic syndrome [6]. This is in line with another human study which also indicated the highest level of *IL10* in the overweight group of objects, compared to non-obese and overweight individuals [9]. However, contradictory findings regarding the cytokine levels exist [10]. There is also some evidence that low *IL10* is not associated with the metabolic syndrome [7].

Our understanding of obesity has been also based on genetic studies. Genes can influence the development of obesity. From this point of view, obesity can be considered in three broad categories. First-monogenic obesity, when a single gene is affected; second- obesity is only one of the symptoms of congenital genetic disorders; and third-polygenic, caused by the presence of genetic polymorphisms in several genes. The pace of gene discovery for obesity accelerated with an approach that screens whole genomes for associations between genetic variants and a phenotype of interest; namely genome-wide association studies (GWAS). To date, GWAS for BMI, waist-to-hip ratio, and other adiposity traits have recognised nearly 300 single-nucleotide polymorphisms (SNPs). Therefore, there is a reason to hope that these discoveries will eventually lead to new preventive measures and therapeutic agents to target obesity [11]. With regards to *IL10*, at least 49 *IL10*-associated polymorphisms have been reported. In addition, a larger number of polymorphisms has been recorded in single nucleotide polymorphism (SNP) databases [12]. One hypothesis claims that some populations may be genetically susceptible to increased fat storage, which may be beneficial for the periods of starvation, but in developed societies results rather in obesity and diabetes mellitus type 2 [13]. Many candidate genes and polymorphisms have been considered, including variants of the *IL10* genes, involved in inflammatory process and many other mechanisms related to the development of overweight and obesity [14,15].

Until now, the relationship between some polymorphisms in the genes encoding Il1, IL6, IL18RAP and overweight or obesity has been described [16,17,18]. However, little is known regarding the relationship between SNPs of the *IL10* gene and the risk of overweight and obesity in young men. The aim of the present study was to investigate the relationship between SNPs of the *IL10* gene (rs1518110, rs3024491, rs2834167) and the risk of overweight in young men.

## 2. Materials and Methods

### 2.1. Ethics Committee

All participants were recruited from the group of cadets of the Military University in Warsaw. Before the study, volunteers were acquainted with the research methods and protocol. All participants were given an information sheet concerning the study and a consent form, providing all crucial information (purpose, procedures, risks, and benefits of participation in the study). All subjects gave their informed consent to participate. The research was conducted in accordance with the Declaration of Helsinki. The protocol was also approved by the Ethics Committee of the Military Institute of Hygiene and Epidemiology-resolution number 07/2018 issued on 23 February 2018.

### 2.2. Participants

The study included 139 male volunteers aged 19–29 years old. Participants filled out the questionnaire screening for exclusion criteria including past diseases, related injuries and ailments, and the presence of severe and chronic pain of any organ or system, both in the past and currently. General medical examinations were conducted (including electrocardiography (ECG) testing) to confirm good health. The subjects were homogeneous in terms of sex, age, food intake and physical activity. Military students examined ate their meals at the student cafeteria and their physical fitness is essential for their profession. Military University students must pass a fitness test, which is an obligatory test performed once a year to evaluate the physical condition of Polish Army soldiers.

### 2.3. Anthropometric Measurements and Body Composition

Anthropometric measurements and body composition were obtained using standard methods. Height was measured using a portable stadiometer with a precision of 0.1 cm (TANITA HR-001, Tanita Corporation, Japan). Body composition and mass analysis measurements were performed using the TANITA MC-780 analyzer (Tanita Corporation, Japan) according to the procedure specified in the instruction manual. The assessment of BMI values was made in accordance with the criteria set out by WHO (WHO 2000).

Three analyses were conducted. In each of them, all participants (*n* = 139) were divided into two groups: OVER and CONTROL depending on their BMI, FMI and Fat% value (Table 1). The overweight group OVER_BMI_ consisted of people with BMI value ≥ 25.0, while the control group CON_BMI_ consisted of people with BMI lower than 25.0 (CDE 2011). The following formula was used to determine the BMI value: body mass index (BMI) = body weight/height^2^ (kg/m^2^). The experimental group OVER_FMI_ consisted of people with a fat mass index higher than 6, while the people in the CON_FMI_ group had FMI values ≤ 6. The FMI classification scale was introduced by Kelly et al. [16]. The following formula was used in order to calculate the FMI values: fat mass index (FMI) = fat mass/height^2^ (kg/m^2^).

Subjects with fat percentage over 20% were grouped into OVER_Fat_, while the participants with Fat% ≤ 20% were classified into the CON_Fat_ group [19,20].

### 2.4. Genetic Analyses

COPAN swabs were used to collect the subjects’ buccal cells. A High Pure PCR Template Preparation Kit (Roche Diagnostics, Germany) was used to extract Genomic DNA from buccal cells. The extraction was conducted in accordance to the manufacturer’s instructions. Good quantity and quality DNA samples were stored at −20 °C for further analysis. All samples were genotyped using TaqMan^®^ Pre-Designed SNP Genotyping Assays for *IL10* (rs1518110) C___8828802_20, *IL10* (rs3024491) C__15983669_10, *IL10RB* (rs2834167) C__25473700_10 single-nucleotide polymorphisms (SNPs) (Applied Biosystems, USA) on a CFX Connect Real-Time PCR Detection System (BioRad, USA) according to the manufacturer’s instructions and recommendations. The PCR protocol was as follows: 5 min of initial denaturation (95 °C), 40 cycles of denaturation (15 s, 95 °C) and annealing/extension (60 s, 60 °C).

### 2.5. Statistical Analyses

All statistical analyses were conducted using R program with specific R packages (version 2.0-1, The R Foundation for Statistical Computing; https://cran.r-project.org accessed on 12 July 2021). Genotype and allele frequency distributions, in addition to HWE probabilities were calculated with SNPassoc package. Single locus analysis including SNP × SNP interaction was also calculated with SNPassoc package under four genetic models (codominant, dominant, recessive and overdominant). The influence of single alleles on BMI, Fat% and FMI was conducted with PearsonX^2^ test using STAT.package. The haplo.score function from haplo.stats package was used to investigate the association between haplotype combinations and BMI, Fat% and FMI under three different models (additive, dominant and recessive). The level of statistical significance was set at the level of *p* < 0.05.

## 3. Results

The results of the anthropometric analysis and the body composition of the studied groups are presented in Table 1.

Amongst parameters such as age, height, weight, metabolic age, visceral tissue index, Fat (kg), FFM, FFMI, water%, BMI, FMI, Fat% according to the t-test, highly significant differences were found with *p*-value between 0.00 and 0.01 for OVER_BMI_ and CON_BMI_ groups, CON_FMI_ and OVER_FMI_, CON_fat_ and OVER_fat_ simultaneously there were no statistically significant differences found for age and height (*p*-value = 0.12 and 0.49 for BMI partition, *p*-value = 0.07 and 0.16 for FMI partition and *p*-value = 0.27 and 0.42 for Fat mass % partition) for the same groups.

A summary of SNPs for *IL10* and *IL10RB* is provided in Table 2, including genetic variation, chromosomal position and gene location [21,22,23].

All genotype frequencies did not significantly differ from the Hardy-Weinberg equilibrium expectations in the OVER_BMI_ group (*p*-values range 0.48 to 1.00), CON_BMI_ group (*p*-values range 0.53 to 0.80), OVER_Fat_ group (0.13 to 0.61), CON_Fat_ (0.58 to 1.00), OVER_FMI_ (*p*-values range 0.13 to 1), CON_FMI_ (*p*-values range 0.38 to 0.83) as well as the case-control group (*p*-values range 0.31 to 1.0) (Table 3).

The influence of *IL10* polymorphism on body mass and composition was tested in recessive, dominant, codominant and overdominant models. No significant association was found between *IL10* (rs1518110), *IL10* (rs3024491), *IL10RB* (rs2834167) and the BMI value exceeding 25 (Table 4, Table 5 andTable 6), Fat% exceeding 20 and FMI value exceeding 6.

For *IL10* (rs1518110) × *IL10* (rs3024491) influence on BMI, Fat% and FMI haplotype analysis was made. Only haplotypes with frequency over 5% was considered. Most common haplotype was CC (0.28%, _*IL10*_ (rs1518110) A > C, _*IL10*_ (rs3024491) C > A). No significantly association was found in haplotype *IL10* (rs1518110-rs3024491) with the BMI, Fat%, FMI in additive, dominant, recessive models (Table 7).

Gen-Gen interactions were calculated for the same genetic models as for single gene analysis (except overdominant model). No association was found between the SNP-SNP interaction with BMI and FMI values for cases (*IL10* (rs1518110) × *IL10RB* (rs2834167), *IL10* (rs3024491) × *IL10RB* (rs2834167)). Significant association was found for interaction *IL10* (rs1518110) × *IL10RB* (rs2834167) with Fat% value exceeding 20 in codominant (*p* value = 0.03, OR = 0.34, 95% CI 0.08 1.44) (Table 8) and dominant model (*p*-value = 0.03, OR = 0.34, 95% CI 0.08 1.44) (Table 9).

The prevalence of A/G × A/A is almost three times less than A/A × A/A (*IL10RB* × *IL10*) and two times less for the A/G × A/C than for A/A × A/C (*IL10RB* × *IL10*) in OVER _FAT_ group in codominant model. In the same group alleles arrangement, A/G × A/A was almost three times less than for A/A × A/A (*IL10RB* × *IL10*) and one and a half times less for the A/G × A/C − C/C than for the A/A × A/C − C/C (*IL10RB* × *IL10*) in dominant model.

**Figure 1:**
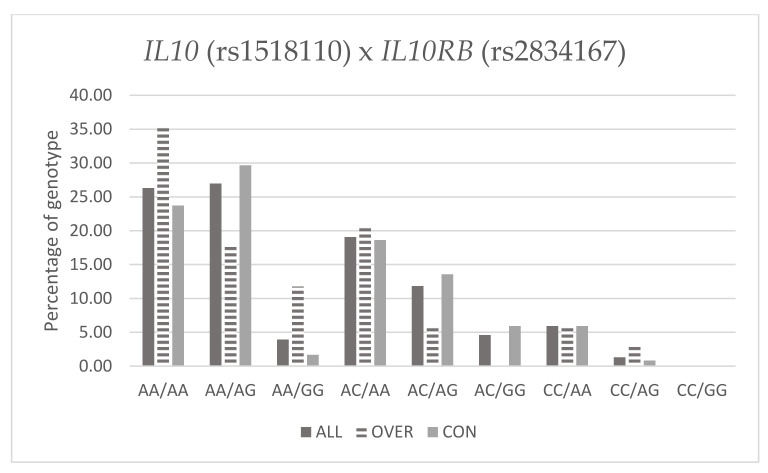
Percentage of genotype *IL10* (rs1518110) and *IL10RB* (rs2834167) with Fat%.

Table 10 and Figure 1 below present frequency of genotypes *IL10* (rs1518110) and *IL10RB* (rs2834167) in Fat% groups.

## 4. Discussion

Our study shows for the first time that there is a correlation between the occurrence of specific polymorphisms of *IL10* and *IL10RB* genes (rs1518110, rs3024491 and rs2834167, respectively) and the possibility of being overweight.

Recent investigations indicate that obesity increases a state of chronic, low-grade inflammation. There is significant interest in the role of adipose tissue macrophages (ATMs) in the inflammatory changes characteristic of obesity. Macrophages show significant heterogeneity in function, depending on local environmental factors that shape their properties and activation state [24]. M1 or “classically activated” macrophages are induced by pro-inflammatory mediators such as LPS and IFNγ. Their activation leads to enhanced pro-inflammatory cytokine production (TNFα, IL6, and IL12) and reactive oxygen species such as NO [19]. M2 or “alternatively activated” have low pro-inflammatory cytokine expression and instead generate high levels of anti-inflammatory cytokines *IL10* and IL1 decoy receptor. One of the markers of M2 is the anti-inflammatory cytokine *IL10*, which is overexpressed in ATMs from lean compared with obese mice [24,25,26].

Some of the studies indicate polymorphisms in *IL10* and *IL10RB* genes as associated with obesity. In our study, significant association was found for interaction *IL10* (rs1518110) × *IL10RB* (rs2834167) with Fat% value exceeding 20 in codominant (*p* value = 0.03, OR = 0.34, 95% CI 0.08 1.44) and dominant model (*p*-value = 0.03, OR = 0.34, 95% CI 0.08 1.44).

We have shown that the chance of being OVER_Fat_ for the A/G × A/A was almost three times lower than for A/A × A/A (*IL10RB* × *IL10*) and two times lower for the A/G × A/C than for A/A × A/C (*IL10RB* × *IL10*).

In addition, it was shown that the chance of being OVER_Fat_ for the A/GxA/A system was almost three times lower than for A/A × A/A (*IL10RB* × *IL10*) and one and a half times lower for the A/G × A/CC/C than for A/A × A/CC/C (*IL10RB* × *IL10*). Our results show a relationship between the frequency of the *IL10* and *IL10RB* genotypes patterns and increased body fat. These results are consistent with hypothesis of inflammation and macrophage recruitment, in which the decrease of inflammatory signals in adipose tissue are caused by retaining M2 macrophages or by converting M1 macrophages to M2. *IL10* may be involved in this mechanism as chronic *IL10* secretion can suppress classically activated M1 macrophages [24].

In our study we demonstrated correlation between body Fat% and specific *IL10* genotype combination. Unfortunately this did not translate into association between those genotypes and BMI. An association of *IL10* with obesity is reported mainly in correlation with ATM and inflammatory balance. Lean adipose tissue is rich in type 2 macrophages and anti-inflammatory cytokines such as *IL10*, obesity changes the balance in favour of a pro-inflammatory milieu, which can lead to the development of insulin resistance and the dysregulation of systemic metabolism. Our result demonstrates unfavourable genotype combination that leads to increase body fat content [27]. 

Reported results regarding the relationship between *IL10* and obesity are conflicting. Esposito et al. [6] demonstrated that circulating *IL10* levels were significantly higher in obese (*n* = 50) than in lean women (median 2.45 vs. 1.2 pg/mL; *p* = 0.04). Increased *IL10* mRNA was found in human subcutaneous adipose tissue from obese patients compared to lean subjects [6]. Opposite results were described by Manigrasso et al., where circulating *IL10* concentration was significantly decreased in obese women (median 1.8 vs. 3.5 vs. 4.1 pg/mL, *p* < 0.0001 in women with android obesity vs. gynoid obesity vs. non-obese women) [10]. No significant change in median *IL10* levels has been observed after body weight reduction or after a physical training program [10,28].

Scarpelli et al. demonstrated that polymorphisms in the *IL-10* promoter associated with low *IL-10* expression are correlated with increased obesity and insulin resistance [29]. Moreover, in obese humans and rodents, adipocytes are significant sources of *IL-10* in obesity. Increased levels of pro-inflammatory cytokines are reported in obesity and this observation is consistent with the hypothesis that a balance exists between pro-inflammatory and anti-inflammatory factors in adipocytes regulating their function [30]. *IL10* rs1518110 and rs3024491 are non-coding variants localized in introns 1 an2 respectively, with no clinical significance, but as we mention in introduction, they may represent a large part of the loci variation due to linkage disequilibrium with other gene polymorphisms [21] *IL10RB* gene encodes *IL10R2*, that is a one of component of *IL10* receptor complex, involved in ligand-mediated assembly inducing activation of STAT3 and STAT3-responsive genes. Associations between *IL10RB* polymorphism and several diseases have been reported [31]. Frodsham et al. identified *IL10RB* as a major susceptibility locus for hepatitis B virus (HBV) persistence, and demonstrated rs2834167 as associated with a risk of HBV persistence [32]. Data indicates *IL10RB* rs2834167 is also associated with Inflammatory Bowel Disease [33] and systemic sclerosis [31]. *IL10RB* rs2834167 codes for a nonsynonymous substitution E47K and is considered to be functional, resulting in premature stop codon and transcript shortening [31].

## 5. Conclusions

To the best of our knowledge this is the first study to evaluate the *IL10* polymorphisms combination in relationship to body fat content. Our data suggest that some genotype combination of *IL10* polymorphisms favour increase body fat and thereby may lead to body mass gain. Thus, it could be more genes from *IL10* family implicated in body fat content. Therefore, more research on this topic needs to be undertaken. Further genes related to overweight should be investigated, including *IL10RA*, which is important in inflammatory signaling and many other mechanisms related to overweight or obesity.

## Figures and Tables

**Table 1:** Anthropometry and body composition of the subjects for BMI, FMI and Fat%.

GROUP (*n* = 139)	CON_BMI_ (*n* = 87)	OVER_BMI_ (*n* = 52)	CON_FMI_ (*n* = 129)	OVER_FMI_ (*n* = 10)	CON_Fat_ (*n* = 116)	OVER_Fat_ (*n* = 23)
Age	22.02 ± 1.86	22.63 ± 2.36	22.10 ± 1.92	24.10 ± 2.95	22.13 ± 1.89	2.83 ± 2.78
Height	179.99 ± 6.20	180.76 ± 6.53	180.07 ± 6.18	183.00 ± 7.60	180.08 ± 6.00	181.26 ± 7.74
Weight	75.30 ± 6.69 *	87.88 ± 7.66 *	78.57 ± 8.13 *	97.23 ± 5.31 *	77.81 ± 7.76 *	90.50 ± 9.25 *
Metabolic age	15.92 ± 4.55 *	26.96 ± 7.86 *	18.50 ± 6.34 *	38.10 ± 2.88 *	17.19 ± 5.25 *	33.57 ± 4.62 *
Visceral Tissue	2.03 ± 0.91 *	4.67 ± 1.80 *	2.64 ± 1.30 *	7.70 ± 0.78 *	2.41 ± 1.14 *	6.00 ± 1.64 *
Fat (kg)	11.02 ± 2.48 *	17.52 ± 4.40 *	12.55 ± 3.43 *	24.42 ± 2.53 *	11.95 ± 3.00 *	20.75 ± 3.92 *
FFM (kg)	64.28 ± 5.44 *	70.36 ± 4.84 *	66.02 ± 5.85 *	72.81 ± 3.85 *	65.87 ± 5.78 *	69.75 ± 6.00 *
FFMI (pkt)	19.82 ± 0.97 *	21.53 ± 0.84 *	20.34 ± 1.18 *	21.78 ± 1.21 *	20.29 ± 1.21 *	21.22 ± 1.10 *
Water%	61.86 ± 2.44 *	58.03 ± 3.25 *	61.02 ± 2.75 *	53.50 ± 1.68 *	61.48 ± 2.49 *	55.42 ± 2.15 *
BMI (pkt)	23.21 ± 1.33 *	26.87 ± 1.56 *	24.20 ± 1.89 *	29.08 ± 1.77 *	23.96 ± 1.81 *	27.52 ± 1.96 *
FMI (pkt)	3.40 ± 0.73 *	5.35 ± 1.25 *	3.86 ± 1.01 *	7.31 ± 0.84 *	3.68 ± 0.89 *	6.30 ± 1.07 *
Fat%	14.55 ± 2.71 *	19.75 ± 3.65 *	15.79 ± 3.25 *	25.09 ± 1.82 *	15.21 ± 2.88 *	22.77 ± 2.38 *

* highly significant differences between groups according to t-test, BMI—body mass index, FMI—fat mass index, FFM—fat-free mass, FFMI—fat-free mass index, Fat—fat percentage, CON—control group, OVER—overweight group.

**Table 2:** The characteristics of single nucleotide polymorphisms for *IL10* and *IL10RB* genes.

Gene	SNP ID	Chromosomal Position	Variation	Gene Location	Functional Consequence
*IL-10*	rs1518110	1:206771516 (GRCh38.p13)	c.A > C	Intron	upstream transcript variant
*IL-10*	rs3024491	1:206771701 (GRCh38)	c.C > A	Intron	upstream transcript variant
*IL10RB*	rs2834167	21:34640788 (GRCh37)	c.A > G, p.Lys(A)47Glu(G)	Exon	missense variant

**Table 3:** The genotype frequencies from Hardy-Weinberg expectations and minor allele frequencies (MAF).

SNP	MAF (%)	ALL	OVER_BMI_	CON_BMI_	OVER_Fat_	CON_Fat_	OVER_FMI_	CON_FMI_
*IL10* (rs1518110)	Allele C (26.26)	0.83	0.48	0.79	0.61	1.00	1.00	0.82
*IL10* (rs3024491)	Allele A (45.68)	0.31	0.56	0.53	0.21	0.58	0.57	0.38
*IL10RB* (rs2834167)	Allele G (29.14)	1.00	1.00	0.80	0.13	0.66	0.13	0.83

MAF—Minor allele frequency, BMI—body mass index, FMI—fat mass index, Fat—fat percentage, CON—control group, OVER overweight group.

**Table 4:** Association analysis of the *IL10* rs3024491 polymorphism with BMI.

		OVER_BMI_ (*n* = 51)	%	CON_BMI_ (*n* = 88)	%	OR	95% CI		*p*-Value	AIC
Codominant	C/C	20	39.2	24	27.3	1.00			0.28	186.2
	C/A	22	43.1	41	46.6	0.64	0.29	1.42		
	A/A	9	17.6	23	26.1	0.47				
Dominant	C/C	20	39.2	24	27.3	1.00			0.15	184.6
	C/A-A/A	31	60.8	64	72.7	0.58	0.18	1.24		
Recessive	C/C-C/A	42	82.4	65	73.9	1.00			0.25	185.4
	A/A	9	17.6	23	26.1	0.61	0.28	1.21		
Overdominant	C/C-A/A	29	56.9	47	53.4	1.00			0.69	186.6
	C/A	22	43.1	41	46.6	0.87	0.26	1.44		
Alleles	C	62	60.78	89	50.57	1.51	0.90	2.57	0.13	
	A	40	39.22	87	49.43					

OR—odds ratio, 95% CI—confidence intervals, BMI—body mass index, CON—control group, OVER—overweight group.

**Table 5:** Association analysis of the *IL10* rs1518110 polymorphism with BMI.

		OVER_BMI_ (*n* = 51)	%	CON_BMI_ (*n* = 88)	%	OR	95% CI		*p*-Value	AIC
Codominant	A/A	28	54.9	48	54.5	1.00			0.64	187.8
	C/A	18	35.3	35	39.8	0.88				
	C/C	5	9.8	5	5.7	1.71	1.84	1.94		
Dominant	A/A	28	54.9	48	54.5	1.00			0.97	186.7
	C/A-C/C	23	45.1	40	45.5	0.99	0.46	6.45		
Recessive	A/A-C/A	46	90.2	83	94.3	1.00			0.37	185.9
	C/C	5	9.8	5	5.7	1.80	0.49	1.97		
Overdominant	A/A-C/C	33	64.7	53	60.2	1.00			0.60	186.5
	C/A	18	35.3	35	39.8	0.83	0.50	6.56		
Alleles	A	74	72.55	131	74.43	0.91	0.51	1.65	0.84	
	C	28	27.45	45	25.57					

OR—odds ratio, 95% CI—confidence intervals; BMI—body mass index, CON—control group, OVER—overweight group.

**Table 6:** Association analysis of the *IL10RB* rs2834167 polymorphism with BMI.

		OVER_BMI_ (*n* = 51)	%	CON_BMI_ (*n* = 88)	%	OR	95% CI		*p*-Value	AIC
Codominant	A/A	26	51.0	44	50.0	1.00			0.97	188.7
	A/G	21	41.2	36	40.9	0.99				
	G/G	4	7.8	8	9.1	0.85	0.48	2.04		
Dominant	A/A	26	51.0	44	50.0	1.00			0.91	186.7
	A/G-G/G	25	49.0	44	50.0	0.96	0.23	3.09		
Recessive	A/A-A/G	47	92.2	80	90.9	1.00			0.80	186.7
	G/G	4	7.8	8	9.1	0.85	0.48	1.92		
Overdominant	A/A-G/G	30	58.8	52	59.1	1.00			0.98	186.7
	A/G	21	41.2	36	40.9	1.01	0.24	2.98		
Alleles	A	73	71.57	124	70.45	1.06	0.60	1.89	0.95	
	G	29	28.43	52	29.55					

OR—odds ratio, 95% CI—confidence intervals, BMI—body mass index, CON—control group, OVER—overweight group.

**Table 7:** Haplotype-based association of *IL10* rs3024491 and *IL10* rs1518110 with BMI, Fat%, FMI.

Haplotype (*IL10* rs3024491, *IL10* rs1518110)		Frequency (%)			Additive (Score = 2.83, *p*-Value = 0.24)		Dominant (Score = 2.65, *p*-Value = 0.45)		Recessive (Score = 2.85, *p*-Value = 0.42)	
		OVER_BMI_ + CON_BMI_	OVER_BMI_	CON_BMI_	Score	*p*-Value	Score	*p*-Value	Score	*p*-Value
C	C	0.28	0.33	0.25	−1.58	0.11	−1.46	0.14	−1.15	0.25
A	A	0.46	0.39	0.49	0.34	0.73	−0.04	0.97	0.91	0.36
C	A	0.26	0.27	0.26	1.42	0.16	1.24	0.21	1.09	0.28
Haplotype (*IL10* rs3024491, *IL10* rs1518110)		Frequency (%)			additive (score = 1.44, *p*-val = 0.49)		dominant (score = 2.51, *p*-val = 0.47)		recessive (score = 1.01, *p*-val = 0.80)	
		OVER_Fat_ + CON_Fat_	OVER_Fat_	CON_Fat_	score	*p*-value	score	*p*-value	score	*p*-value
C	C	0.28	0.35	0.27	−0.94	0.35	−1.33	0.18	−0.24	0.81
A	A	0.46	0.39	0.47	−0.03	0.98	−0.19	0.84	−0.16	0.87
C	A	0.26	0.26	0.26	1.06	0.29	1.56	0.12	0.31	0.76
Haplotype (*IL10* rs3024491, *IL10* rs1518110)		Frequency (%)			additive (score = 1.25, *p*-val = 0.53)		dominant (score = 3.01, *p*-val = 0.39)		recessive (score = 0.16, *p*-val = 0.98)	
		OVER_FMI_ + CON_FMI_	OVER_FMI_	CON_FMI_	score	*p*-value	score	*p*-value	score	*p*-value
C	C	0.28	0.4	0.27	−1.58	0.11	−0.66	0.51	−0.91	0.36
A	A	0.46	0.4	0.46	0.34	0.73	−0.51	0.61	−0.24	0.81
C	A	0.26	0.2	0.27	1.42	0.16	1.17	0.24	−0.01	0.99

NA—not applicable, BMI—body mass index, FMI—fat mass index, Fat—fat percentage, CON—control group, OVER—overweight group.

**Table 8:** Association analysis of the *IL10RB* rs2834167 × *IL10* rs1518110 interaction with Fat% (codominant model).

		*IL10*_rs1518110															
		A/A					A/C					C/C					
		OVER_Fat_	CON_Fat_	OR	95% CI		OVER_Fat_	CON_Fat_	OR	95% CI		OVER_Fat_	CON_Fat_	OR	95% CI		*p*-Value
*IL10RB* rs2834167	A/A	7	27	1.00	NA	NA	6	22	1.05	0.31	3.59	1	7	0.55	0.06	5.25	0.03
	A/G	3	34	0.34	0.08	1.44	2	16	0.48	0.09	2.61	1	1	3.86	0.21	69.67	
	G/G	3	2	5.79	0.80	41.61	0	7	0.00	0.00	NA	0	0	NA	NA	NA	

OR—odds ratio, 95% CI—confidence intervals; NA—not applicable, Fat—fat percentage, CON—control group, OVER—overweight group.

**Table 9:** Association analysis of the *IL10RB* rs2834167 × *IL10* rs1518110 interaction with Fat% (dominant model).

			*IL10* rs1518110										
			A/A					A/C-C/C					
			OVER_Fat_	CON_Fat_	OR	95% CI		OVER_Fat_	CON_Fat_	OR	95% CI		*p*-Value
*IL10RB* rs2834167	A/A		7	27	1.00	NA	NA	7	29	0.93	0.29	3	0.03
	A/G		3	34	0.34	0.08	1.44	3	17	0.68	0.15	3	
	G/G		3	2	5.79	0.80	41.61	0	7	0.00	0.00	NA	

OR—odds ratio, 95% CI—confidence intervals; NA—not applicable, Fat—fat percentage, CON—control group, OVER—overweight group.

**Table 10:** Percentage of genotype *IL10* (rs1518110) and *IL10RB* (rs2834167) with Fat%.

*IL10* rs1518110	*IL10RB* rs2834167		OVER_Fat_+ CON_Fat_	OVER_Fat_	CON_Fat_
AA	AA	AA/AA	26.32	35.29	23.73
AA	AG	AA/AG	26.97	17.65	29.66
AA	GG	AA/GG	3.95	11.76	1.69
CA	AA	AC/AA	19.08	20.59	18.64
CA	AG	AC/AG	11.84	5.88	13.56
CA	GG	AC/GG	4.61	0.00	5.93
CC	AA	CC/AA	5.92	5.88	5.93
CC	AG	CC/AG	1.32	2.94	0.85
CC	GG	CC/GG	0.00	0.00	0.00

Fat—fat percentage, CON—control group, OVER—overweight group.

## Data Availability

The data presented in this study are available upon request from the corresponding author. The data are not publicity available due to ethical reasons.
